# Cholera in Rochester during the Summer of 1852

**Published:** 1853-02

**Authors:** 


*Cholera in Rochester during the Summer of* 1852.—From a "Report of the Board of Health on Cholera as it appeared in Rochester, New York, in 1852," we take the following extract, which gives an account of the successive occurrence of cases, the locations and their sanitary conditions, the number of cases during each month, fatality, etc. The circumstances connected with the rise and progress of this epidemic in different places should be placed on record with a view to the accumulation of facts which may prove useful in future investigations of the causation of the disease. — Ed. Buffalo Med. Journal.

*Cholera — Its History and Progress.* — Having described the sanitary condition of the city prior to its appearance, and during the first few cases of cholera, and the means provided for the relief of the sick, the Board proceeds to give as detailed and accurate an account of the epidemic from its commencement to its termination, as the data in its possession will permit—premising, that many of the reports of physicians were very defective in the information communicated—and not a few of them, it is feared, not altogether accurate in their details.

On the 6th of June, Dr. Swinburne was called to prescribe for John Hart, an Irish laborer, residing in an old house on Factory street. The man had bilious vomiting and purging. About 9 o'clock in the evening of the following day, June 7th, his attention was called to the patient's daughter, Margaret Hart, aged 10 years—who had been seized with vomiting and purging some hours before—and on examining her, she was found pulseless, eyes sunken, extremities cold—skin of a leaden hue—discharges, watery; and the startling fact was apparent, that she had cholera, and was then in a state of hopeless collapse. She died at 4 o'clock on the following morning, June 8.

This case was reported to a member of this Board, who, in company with Dr. Swinburne, on the following day, June 9, examined the premises. The house was an old rookery—without a cellar, and presented inside the usual appearance of the residences of the poorer classes of laborers with the usual smell—but nothing very remarkable—the father still lying sick in a small illy-ventilated room adjoining the kitchen, which also answered the purpose of a dining-room, and probably of a dormitory also at night. The yard in rear of the house presented nothing objectionable—it was in good condition, but at the west end of the house a small pile of rubbish was discovered, and almost adjoining the house, a small pen, containing two small pigs. The condition of things outside the house would bear a very favorable comparison with that inside. In front and near the door, was an opening into the main sewer—into which the slops were thrown—but the water was seen running clear, with a free current—and no smell was discernable from the source. The premises were again inspected a few days afterward, but nothing new was discovered. The health inspectors were in the mean time put on the alert.

The next case that presented itself, was that of James Ward, aged 21 years, who sickened and died in the old "Factory Block," June 16; and three days afterward, his sister, Catharine Ward, aged 38 years, who occupied the same apartments, died also. On the following day, June 20, Patrick Enright, residing in the same block, sickened, but recovered, June 22. John Ferguson, aged 20 years, sickened and died on the 23d. Mary Dooley, in the same block, also, sickened the same day, and recovered. In the night of the 24th, William Cashmere, an English laborer, residing in the same block, sickened, and died on the 26th; and his son, aged five years, died on the following night. These were all the cases reported to the Board from the 8th to 26th June, inclusive: five in the Factory Block, and one in the old house, first named, and separated from the former only by a narrow court-yard.

The above deaths—the first on the 8th, and the others between the 16th and 26th June—occurring all in one locality, impressed the Board with a deep conviction, that there must be some great defect in the sanatary arrangements on the premises. Accordingly, a thorough search was made, but nothing was discovered to account for so great a mortality, except it were over-crowding; and on a careful inquiry made in the premises, on the 22d June, it was ascertained, that there were in the "Old Factory Block," properly so called,

| | | |
|---|---|---|
| Adults, . . . . . . . . . | 31 | |
| Children, . . . . . . . . | 25 | |
| | | 56 |
| And in the block adjoining, on Mill and Factory streets, | | |
| Adults, . . . . . . . . . | 12 | |
| Children, . . . . . . . . | 14 | 26 |
| Amounting in all, including old and young, to . | | 82 |

individuals, living in close, illy-ventilated rooms, and as a necessary consequence, not kept in the best condition: but as a general thing, the premises outside the buildings, with the exception under Higgins' Block, already mentioned, were not in bad condition. But all the buildings were old and decayed, and as might well be supposed, even in a condition to produce the poison which proved so fatal in its effects.

On the 27th and 28th June, two children, Ellen and Allice Hickey, aged two and four years, died on Cayuga street. These make up eight cases in all, which occurred in the month of June—the same number mentioned by J. Moran, in his report to the Mayor, viz: June 15th, J. Ward, aged 24 years, State street; Mary Moran, aged 20 years, State street; John Ward, aged 24, State street; June 23, D. Ferguson, Frank street, aged 28; June 24, Patrick McGuire, aged 18, Fish street; June 28, J. O'Mera, aged 2 years, Cayuga street; and two children of Mr. Tuckey, on the same street, aged 4 and 6 years. It will be noticed, that the name of J. Ward is mentioned twice, while that of Margaret Hart, who died on the 8th, and those of William and Solomon Cashmere, are omitted altogether. D. is substituted for *John* Ferguson, whose residence is put down as Frank street; and the name of Mary Moran, was probably intended for that of Catharine Ward. It is singular enough, that so many mistakes should occur in recording the names of only eight persons—the ages of them being certainly wrong—the streets also wrong; and we have a right to infer, that the others are wrong also. The errors, which are known to be so, should at least put us on our guard, and should warn us not to place too high an estimate on the remainder of this and other similar reports—the propriety of which will be more clearly seen hereafter.

On the 29th of June, John Hickey, the father of Ellen and Allice Hickey, sickened, and died, 3d July. He was very intemperate; was absent from home from the 26th to 29th June; said he ate nothing while absent; slept out doors, and returned home, sick. It is reasonable to suppose, that his family was poorly provided for, and that the children were poorly fed. No other cause was known for the attack. The only thing discovered about the premises that could affect the health unfavorably, was an open spring, and a drain leading from it in one corner of the garden, a few feet from the house.

The ground, aside from this, was dry, loomy, and clean; cellar, dry; and the house in tolerable condition, for that of a drunken man. John Hoolihan, a boarder in the next house, said to be of good habits, died the same day; no cause for the attack being known.

No other cases are known to have occurred until the 9th and 10th of July, when two fatal cases took place on Furnace street; the mother, 35, and one child, 5 years old; the former leaving behind her an infant of three weeks, which still survives. Another child belonging to the same family, aged 3 years, sickened on the 13th, and recovered.

On the 18th, several fatal cases occurred on an alley opposite Gorham street, east side of the river; and also on Mill street, west side; and soon after, several fatal cases occurred in Taylor's Block, on Fish street—one in Lester's Block, on State street; three in Congress Hall, and one in the Jail. Fatal cases also occurred on State, Buffalo, Furnace, Mumford, Front, Water, Exchange, Court, Delevan, and Clay streets; and also on Brown, Pine, and Pindle alleys. During this time, until the 30th, inclusive, from one to two or three deaths occurred daily in the localities above named.

On the 31st July, about fifteen new cases occurred—nearly all of which proved fatal. Seven of these fatal cases occurred in Lester's Block, on State street; and on the following day, six other fatal cases occurred in the same block. The utmost consternation and alarm were produced by this sudden increase of the epidemic, not only in the block itself and its immediate neighborhood, but throughout the city. The block was very soon emptied of the principal part of its fear-stricken occupants, who fled to other parts of the city, and to the country, for safety; and several were overtaken in their flight, or after their arrival at their supposed places of safety, and fell the unhappy victims of their ruthless pursuer. In the course of one short week, twenty-five individuals, old and young, who had tenanted this fatal block, became the tenants, each, of a peaceful grave—two whole families having been nearly swept away; out of fourteen belonging to these families, only four survive—each family having lost five of its members, the parents of each being included.

In no other locality did the epidemic show itself with such perfect violence—but it was manifest, that it had assumed an increased degree of potency in other parts of the city—and the number of fatal cases increased from two or three, to ten or twelve each day. This, however, continued only a very few days—the number in the course of two weeks being reduced to less than one-half, and varying from 3 or 4 to 7; rarely after the 4th of August, exceeding the last number.

On the 2d of August, Moses B. Seward, Esq., a gentleman highly esteemed and respected, fell a victim to the scourge; and on the 6th was followed by Dr. J. J. Treat, whose zeal to relieve the sufferings of those who relied upon his skill, in the hour of their extremity, carried him beyond the just limits of prudence; and, heedless of his own safety, he sacrificed himself in endeavoring to relieve others.

On the 25th, Dr. William Bell, long known here as a physician, also fell a victim to the fell destroyer. His health had been long impaired, and for several days prior to the fatal attack, he had suffered from diarrhœa, which he had either neglected, or for the cure of which he had relied too explicitly on a supposed specific.

On the 15th of August, the epidemic broke out in Sherman's Block, on

Ford street; and in the course of twelve days, ten individuals became its victims.

During the first four days in August, 35 deaths were reported to the Board; and during the next four days, 28 more, or 63 deaths in eight days; averaging nine deaths per day. During the next eight days, 43 more deaths were reported—the whole number for the first sixteen days being 106. During the next fifteen days, 63 more deaths were reported—the whole number for the month being 169.

During the first fifteen days in September, 70; and during the remainder fifteen, 23 more deaths were reported; amounting to 93 during the month. They occurred on the streets already named, and also on Romain, Clinton, Jackson, St. Joseph, Riley, Union, Court, Adams, Jay, Martin, Strong, Bay, and other streets—showing very clearly, that the epidemic cause, or influence, or constitution of the air, had reached every part of the city; and the fact, that a considerable number of cases had appeared, not only in the remote parts of the city, but in the neighboring country around for several miles in extent, and among individuals, who had had no communication with the city, shows beyond all doubt, that it had become pretty generally diffused apparently without regard to any known local causes.

On the 22d of this month, Dr. D. C. Phelps, who resided on Buffalo street, and who had been a resident of the city for about three years, fell a victim to the epidemic. He was an active young man, a native of Ireland—belonged to the Eclectic School, and was thus early cut off, almost in the outset of his professional career.

During the early part, and up to the 18th of October, thirteen deaths took place; among which were those of George F. Martin, a native of England, a man of the most temperate and correct habits, who resided on King street—one of the most cleanly in the city—and his daughter, seven years of age—both of whom died on the 5th, after a few hours' illness. The daughter was first attacked, without any known cause. The attack of the father was apparently produced by fear and excitement, consequent upon seeing the hopeless condition of a beloved child. Neither of them had been guilty of any dietetic imprudence. The house and premises were clean, and in good condition; the cellar, dry and free from smell. Nothing could be discovered in or about the premises, likely to deteriorate the health, or cause the attack. But it was ascertained, on inquiry, that a drain from the cellar communicated with the sewer on the street—one of the tributaries to the Platt street sewer—and although no smell could be discovered coming through this drain, it is nevertheless possible that the poison may have found its way through this avenue. Five weeks previously, a fatal case, that of Mr. Osborne, had occurred in the next house, on the same street.

On the first of November, a person was brought to the jail, who had been unwell for several days—and shortly after died of cholera. A fellow-prisoner, in feeble health, 83 years of age, on being made acquainted with the fact, became panic-struck, and was immediately seized with cholera, and died also. These are the last deaths which are known to have taken place in the city—the last case being in singular contrast with that of the first—a child of ten years.

Owing to the causes already enumerated, it is impossible to state with entire accuracy, the number of deaths which have occurred from cholera. Much pains have been taken and much time expended to ascertain the exact number; but an approximation to correctness is all that has been accomplished. To the list reported by physicians, have been added three cases which were well ascertained from reliable health officers, and from personal inquiries among surviving relatives and friends; and a careful comparison has been made with the reports made to the Mayor by the city sexton or undertakers.

The statistics, showing the number of deaths in each of the months, have already, in part, been anticipated. But those already shown were then only reported to the Board. That the entire subject—not the least interesting—may be the more easily and fully comprehended, the whole, although some repetition may become necessary, will be presented in a single view, and in different aspects.

During the month of June, that is, from the 8th to the 28th, eight deaths were reported, and one has since been ascertained—say,

| | | |
|---|---|---|
| From the 3d to the 30th of July, inclusive, the number of deaths, was . . . . . . . . | 61 | |
| Ditto, on the 31st July, . . . . . . | 17— | 78 |
| The number of deaths on the 1st August, was 12, and during 1st week, . . . . . . . . | 66 | |
| Do. 2d do., . . . . . . . . | 33 | |
| Do. 3d do., . . . . . . . . | 32 | |
| And during the remaining ten days, . . . . . . | 49— | 180 |
| During the first half of Sept., the number of deaths was, . | 112 | |
| " 2d half do. do. | 26— | 138 |
| During the month of October, there were 13, and in Nov., 2 deaths, . . . . . . . . | | 15 |
| The whole number of deaths being, . . . . . | | 420 |

It will be seen that the greatest number of deaths, on any single day, occurred on the 31st July, viz., 17; and on the following day, 12; averaging $14\frac{1}{2}$ for each day.

The average number of deaths for July, was a fraction over $2\frac{1}{2}$; that for August, 5.8, or a portion over $5\frac{3}{4}$; and for September, 4.60, or a little over four and a half.

